# P025. Two-year follow-up with OnabotulinumtoxinA for chronic migraine: a real life evaluation of 113 patients

**DOI:** 10.1186/1129-2377-16-S1-A182

**Published:** 2015-09-28

**Authors:** Antonio Santoro, Matteo Tanzi, Anna Miscio, Massimiliano Copetti, Maurizio Leone

**Affiliations:** IRCCS, Ospedale Casa Sollievo della Sofferenza, Department of Neurology, San Giovanni Rotondo, (FG) Italy; IRCCS, Unit of Biostatistics, Ospedale Casa Sollievo della Sofferenza, San Giovanni Rotondo, (FG) Italy

## Background

OnabotulinumtoxinA (Botox^®^, Allergan) has shown its efficacy in chronic migraine (CM) in two phase III studies and up to 5 injection cycles [1]. However, few studies have been published based on its real life efficacy and few data are available on its efficacy beyond the 5^th^ cycle of treatment [2].

## Objective

To assess the real life efficacy of 155U-195U OnabotulinumtoxinA in CM patients in order to retrospectively investigate the benefits of such treatment and observe if the efficacy is sustained after one year.

## Methods

We reviewed the charts of 134 patients treated with OnabotulinumtoxinA who received up to 9 cycles. Patients were injected regularly with a 3-month (±10 days) interval. They were assessed for headache days and hours, intensity of pain by Visual Analogue Scale (VAS), number of any acute drug intake. Photophobia, phonophobia, osmophobia, nausea were assessed as well. The results were also analysed based on the CM onset.

## Results

Since approval, we have treated a total of 134 patients. We collected the data of 113 CM patients (mean age 48 y.o.; 76% women) who represent the ones showing any response during the first two treatment cycles. Already after cycle two, those who were responders, showed a high decrease vs the baseline as follows: 54% in headache days reduction (from 24.1 to 11.1); 64% in headache hours (from 552.8 to 199). Also, pain intensity dramatically decreased 21% (from 9.6 to 7.6) and correspondingly any drug intake went from an average of 51.65 to 16 tablets/month. In the case of those patients also taking i.v. drugs, these had been totally suspended from the second cycle since there was no need. Thirty-seven patients had been treated longer than one year and up to 9 cycles confirming an increasing improvement over time (Table 1). No difference in efficacy was recorded comparing patients suffering from CM from 5 up to 20 years.

**Table 1:** Table 1

Time in months	Headache Days	% of Reduction vs Baseline
Baseline	24.1 (22.1 - 26.3)	
3	16.4 (14.8 - 18.1)	32
6	11.1 (9.7 - 12.7)	54
9	7.5 (6.3 - 9.1)	69
12	5.1 (4.0 - 6.5)	79
15	3.5 (2.6 - 4.7)	85.5
18	2.4 (1.7 - 3.3)	90
21	1.6 (1.1 - 2.4)	94.4
24	1.1 (0.7 - 1.7)	95.4

## Conclusions

Our real life experience demonstrated the efficacy and tolerability of the OnabotulinumtoxinA responders already after the first treatment cycles. Overtime, not only a sustained efficacy was observed but also a favorable trend of improvement with no significant adverse events. Moreover, our analysis confirmed that the efficacy outcomes were not affected by the CM onset thus allowing us to assume that OnabotulinumtoxinA can be considered a valuable first line treatment to allow more patients to benefit earlier and more consistently from this therapeutic option.

Written informed consent to publish was obtained from the patient(s).
